# Ultrasound evaluation of the penis

**DOI:** 10.1590/0100-3984.2016.0152

**Published:** 2018

**Authors:** Maitê Aline Vieira Fernandes, Luis Ronan Marquez Ferreira de Souza, Luciano Pousa Cartafina

**Affiliations:** 1 MD, Radiologist, Universidade Federal do Triângulo Mineiro (UFTM), Uberaba, MG, Brazil.; 2 MD, Radiologist, Associate Professor in the Department of Radiology and Diagnostic Imaging of the Universidade Federal do Triângulo Mineiro (UFTM), Uberaba, MG, Brazil.; 3 MD, Urologist, Member of the Clinical Staff of the Urology Department of the Universidade Federal do Triângulo Mineiro (UFTM), Uberaba, MG, Brazil.

**Keywords:** Penis, Ultrasonography, Ultrasonography, Doppler, color, Ultrasonography, Doppler, duplex, Penile diseases

## Abstract

Ultrasound is an excellent method for the study of penis. In this article, using a critical review of the literature and teaching files, we present examples of the major findings in the ultrasound routine, focusing on trauma, priapism, Peyronie's disease, and erectile dysfunction.

## INTRODUCTION

Ultrasound is an imaging modality that, in addition to being well tolerated and widely available, is considered an excellent method for the evaluation of many penile diseases^(1)^. Penile trauma, priapism, Peyronie's disease, and erectile dysfunction are some of the conditions in which penile ultrasound finds significant applicability.

Currently, linear transducers have a maximum frequency of 12-15 MHz, which increases the definition on ultrasound images. Although penile evaluation is usually performed when the penis is flaccid, in some situations, such as in cases of erectile dysfunction, the examination should be performed during erection, by injection of vasoactive drugs into the corpora cavernosa^(1)^. The penis should be positioned in the anatomical position (over the abdomen) and evaluated in the transverse and longitudinal directions, from the glans toward the base of the penis^(2)^. In specific situations, such as in the evaluation of Peyronie's disease, the penis should also be positioned on a towel/sheet or on the testicular sac, with a lateral approach.

## ANATOMY AND PHYSIOLOGY

**Figure 1:**
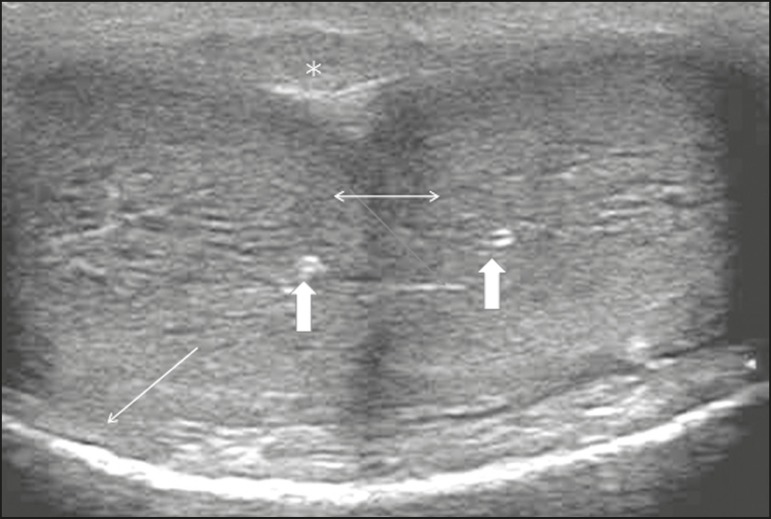
Normal penile anatomy, ventral and cross-sectional views showing two hypoechoic images corresponding to the corpora cavernosa (two-headed arrow), with their respective cavernous arteries (thick arrows), with an echoic line that surrounds them and corresponds to the tunica albuginea (thin arrow). Note the corpus spongiosum (asterisk) adjacent to the corpora cavernosa.

**Figure 2:**
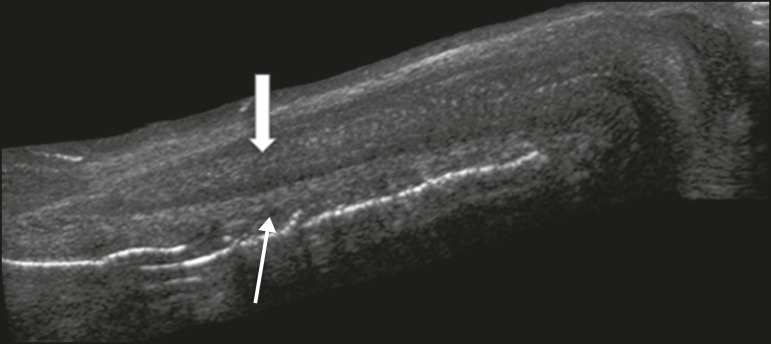
Longitudinal section of the penis under right lateral access. Note the cylindrical structure, corresponding to the corpus cavernosum (thick arrow), in the superior portion and the corpus spongiosum (thin arrow), adjacent to the corpus cavernosum, in the inferior portion.

The corpora cavernosa are homogeneous and relatively hypoechoic cylindrical structures^(2)^ lined with tunica albuginea, a thin membrane that has a thickness of approximately 2 mm when the penis is flaccid and 0.25 mm when it is erect^(3)^. The corpus spongiosum, a ventral, medial body that is more echoic than the corpora cavernosa, is also covered by the tunica albuginea and contains the urethra. As can be seen in Figures 1 and 2, it is more dilated and prominent in its proximal segment, known as the bulb, and in its distal segment, constituting the glans^(2)^. Buck's fascia is superficial to the tunica albuginea and covers all of the structures described.

**Figure 3:**
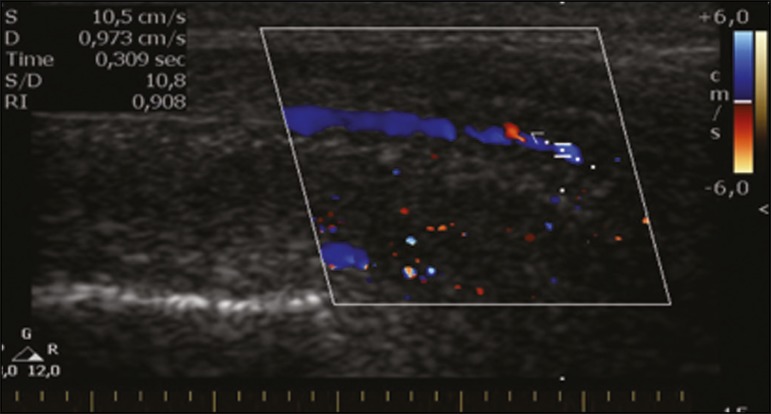
Longitudinal, ventral ultrasound of the penis, with pulsed mode and color Doppler. Normal flow pattern of the cavernous artery in a flaccid penis.

Venous drainage is performed by the deep and superficial dorsal veins of the penis. The dorsal arteries of the penis are located adjacent to the deep dorsal vein and a cavernous artery is located in the center of each corpus cavernosum. On color Doppler, the cavernous arteries present single phase flow. In the flaccid penis (Figure 3), the normal cavernous arteries show a systolic peak between 11 and 20 cm/s^(1)^. At the beginning of erection, the systolic and diastolic flows undergo progressive increases. When vein occlusion begins, the diastolic flow decreases progressively, and once stiffness is established, it becomes negative^(1)^.

## PENILE TRAUMA

Penile trauma can result from a blunt or penetrating injury, the latter being rarely investigated by imaging methods, almost always requiring immediate surgical exploration^(2)^. In the erect penis, trauma results from stretching and narrowing of the tunica albuginea, which can undergo segmental rupture of one or both of the corpora cavernosa, constituting a penile fracture.

**Figure 4:**
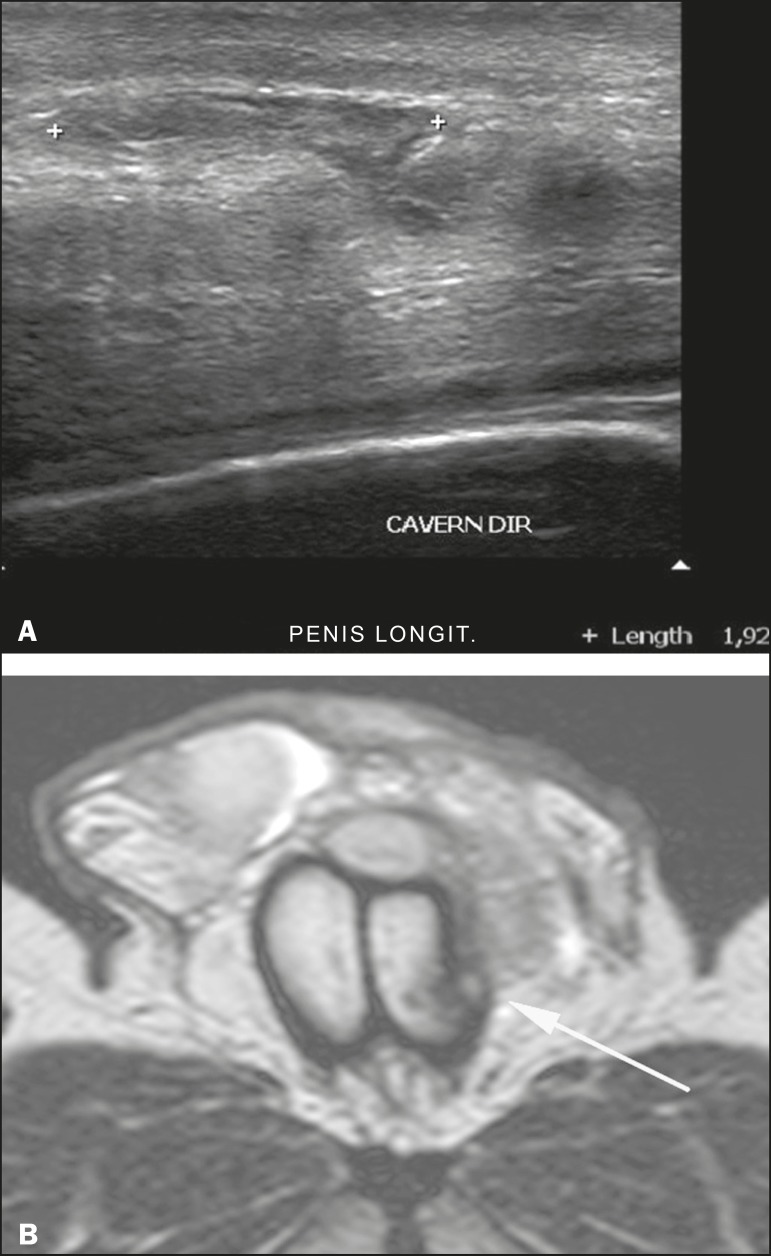
**A:** Ultrasound of the penis, right lateral view. Longitudinal section showing rupture of the tunica albuginea with an adjacent 1.92 cm hematoma (between calipers), due to trauma. **B:** Axial T2-weighted turbo spin-echo magnetic resonance imaging scan showing left-sided discontinuity of the tunica albuginea (arrow), secondary to fracture.

In the ultrasound examination, a lesion of the tunica albuginea presents as an interruption in (loss of continuity of) the echoic line representing it (Figure 4). Small, moderate, or broad hematomas demonstrate the extent of that discontinuity^(4)^. Intracavernous hematomas, sometimes without the presence of a tunica albuginea fracture, can be observed when there is a lesion of the smooth muscle of the trabeculae surrounding the sinusoid spaces or the subtunical venular plexus^(3)^.

In 10-15% of penile traumas, there can be an accompanying urethral lesion^(4)^. When blood is observed in the urethral meatus, contrast-enhanced evaluation of the urethra is necessary. In cases in which the ultrasound findings are inconclusive, the use of magnetic resonance imaging can facilitate the diagnosis^(4)^ and is recommended by various authors^(3,4)^.

## PRIAPISM

Priapism is defined as a painful and prolonged penile erection, with or without sexual stimulation^(4)^. Color Doppler ultrasound is the imaging method of choice for the investigation of priapism, because it is noninvasive, widely available, and highly sensitive^(4)^. By means of this method, it is possible to diagnose priapism and differentiate between its low- and high-flow forms.

Low-flow (ischemic) priapism is a urologic emergency in which there is inadequate venous drainage, resulting in hypoxia, ischemia, and tissue acidosis, which can in turn result in fibrosis and erectile dysfunction. A number of causes have been described, including sickle cell anemia (most common in children), leukemia, and other blood dyscrasias (such as thalassemia and multiple myeloma), and the use of various licit or illicit drugs, as well as neoplasms. On Doppler, the flow in the cavernous arteries is reduced or absent^(4)^. As the condition progresses, there is an increase in echogenicity of the corpora cavernosa, attributed to tissue edema. Eventually, changes in the echotexture of the corpora cavernosa can be observed due to the fibrotic transformation generated by tissue anoxia^(1)^.

**Figure 5:**
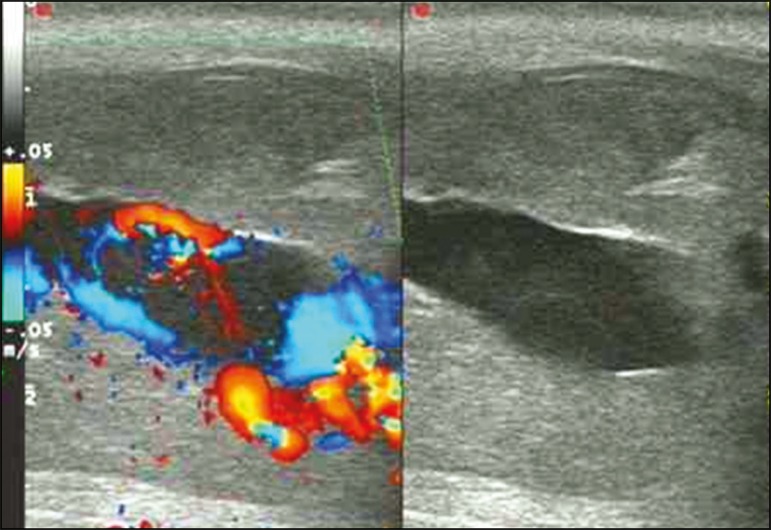
Color Doppler ultrasound demonstrating a hypoechoic collection that corresponds to hematoma with arteriovenous fistula secondary to traumatic injury of the penis due to impact with bicycle handlebars, resulting in high-flow priapism.

High-flow priapism is not considered an emergency, because patients are at lower risk of permanent sequelae^(4)^. It is associated with blunt trauma to the perineum or penis, with laceration of the cavernous artery, which can generate an arterial-lacunar fistula. In the Doppler study, one can observe normal or increased, turbulent blood flow in the cavernous arteries. The area surrounding the fistula presents a hypoechoic, irregular lesion in the cavernous tissue^(4)^, as depicted in Figure 5.

## PEYRONIE'S DISEASE

Peyronie's disease is characterized by fibrotic thickening of the tunica albuginea, which can lead to curvature of the penis and difficulty in achieving an erection^(5)^. Penile fibromatosis (Peyronie's disease) is marked by the formation of fibrous plaques identified as areas where the tunica albuginea is thickened^(1)^. Although plaques are more common on the dorsum of the penis, they can also be seen on the ventral face, lateral face, or septum.

**Figure 6:**
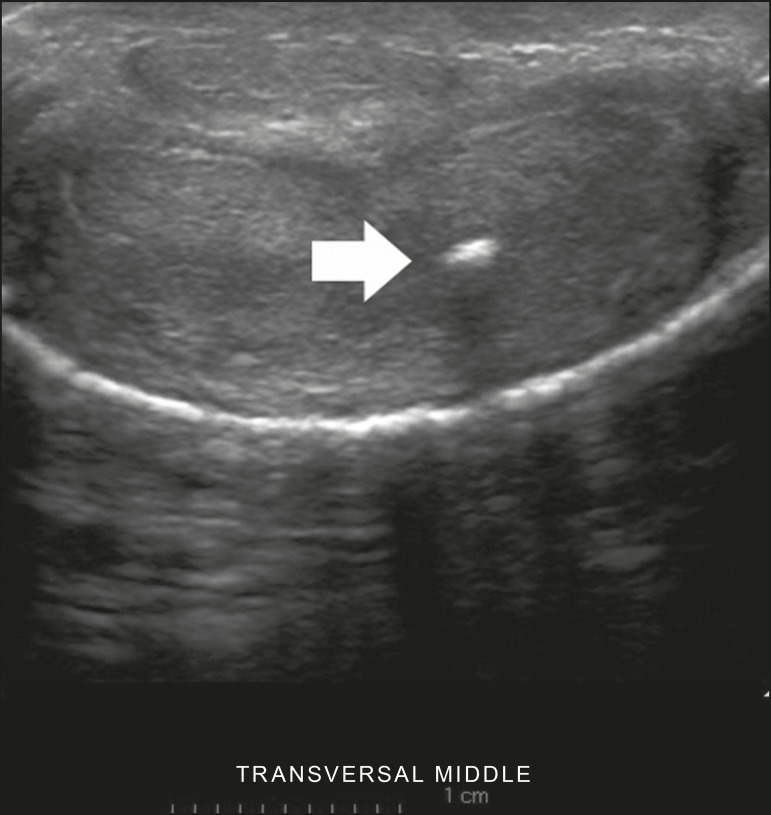
Transverse ultrasound of the penis, in a ventral view, in the middle portion of the penis. Note the echoic image with posterior acoustic shadowing, corresponding to calcification (arrow), in the left corpus cavernosum.

**Figure 7:**
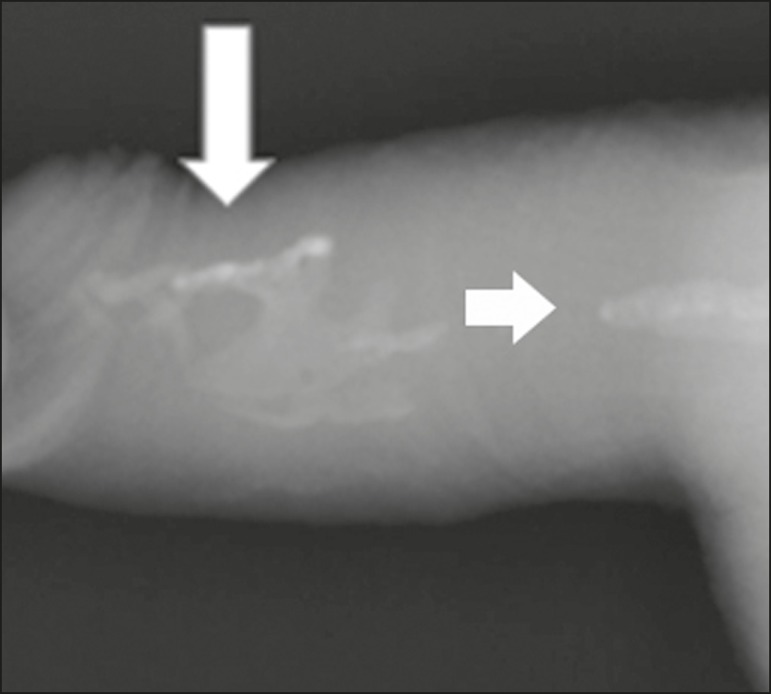
Conventional X-ray, penetrating the soft parts of the penis, showing radiopaque images that correspond to calcifications in the corpora cavernosa (arrows).

The typical finding on ultrasound is hyperechoic focal thickening of the tunica albuginea. Due to associated calcifications, the imaging of patients with Peyronie's disease shows acoustic shadowing^(5)^, as illustrated in Figures 6 and 7. Less common findings, attributed to earlier stages of the disease (still mild fibrosis), are hypoechoic lesions with focal thickening of the paracavernous tissues, echoic focal thickening of the tunica without posterior acoustic shadowing, retractile isoechoic lesions with posterior attenuation of the beam, and focal loss of the continuity of the tunica albuginea.^(5)^. In the Doppler study, increased flow around the plaques can suggest inflammatory activity and the absence of flow can suggest disease stability^(6)^. Ultrasound is useful not only for the identification of lesions but also to determine their relationship with the neurovascular bundle^(1)^. Individuals with Peyronie's disease can present with erectile dysfunction, often related to venous leakage, due to insufficient drainage at the site of the plaque^(5)^.

## ERECTILE DYSFUNCTION

Most cases of erectile dysfunction of organic causes are related to changes in blood flow in the corpora cavernosa, represented by occlusive artery disease, most often of atherosclerotic origin, or due to failure of the veno-occlusive mechanism^(7)^. Preceding the ultrasound examination with Doppler, the penis must be examined in B mode, in order to identify possible tumors, fibrotic plaques, calcifications, or hematomas, as well as to evaluate the appearance of the cavernous arteries, which can be tortuous or atheromatous.

**Figure 8:**
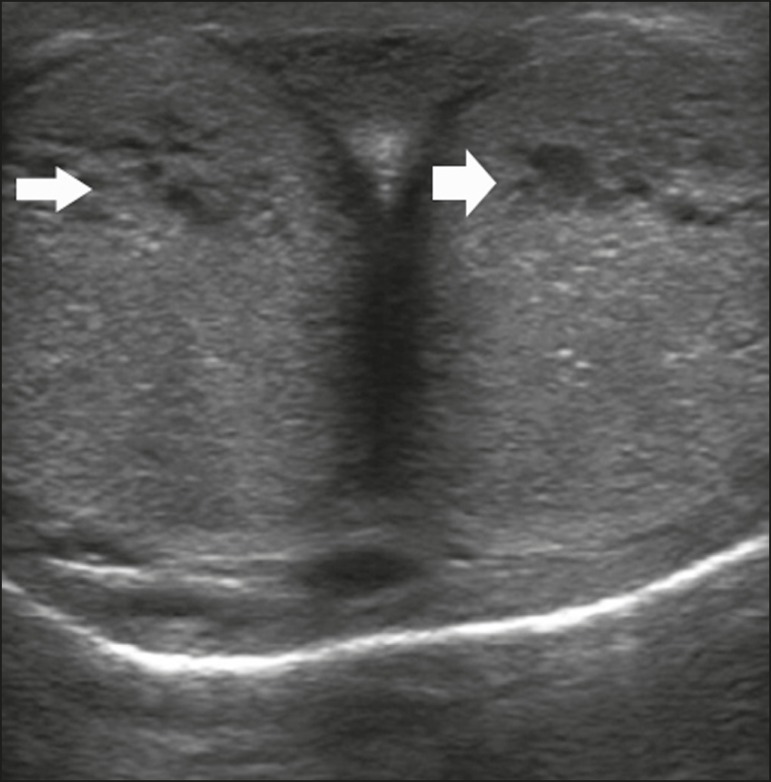
Transverse ultrasound image, ventral view of the penis. Image obtained after induction of an erection, 15 min after injection of prostaglandin E1, showing dilated sinusoids (arrows).

Erection can be induced by injecting 10-20 µg of prostaglandin E1, with evaluations of the arterial flow every five minutes for 25-30 min (Figure 8). The use of prostaglandin E1 is contraindicated in patients with a predisposition to priapism (e.g., those with sickle cell anemia), as well as in those with an anatomical deformity of the penis or a penile implant. Phentolamine (2 mg) is often added. Visual and tactile stimulation produces better results^(8)^. Some authors recommend the use of oral medication (sildenafil citrate, 50 mg) to replace the injectable drug(s) in cases of contraindications, although the efficacy of such medication is controversial in the literature^(9,10)^.

**Figure 9:**
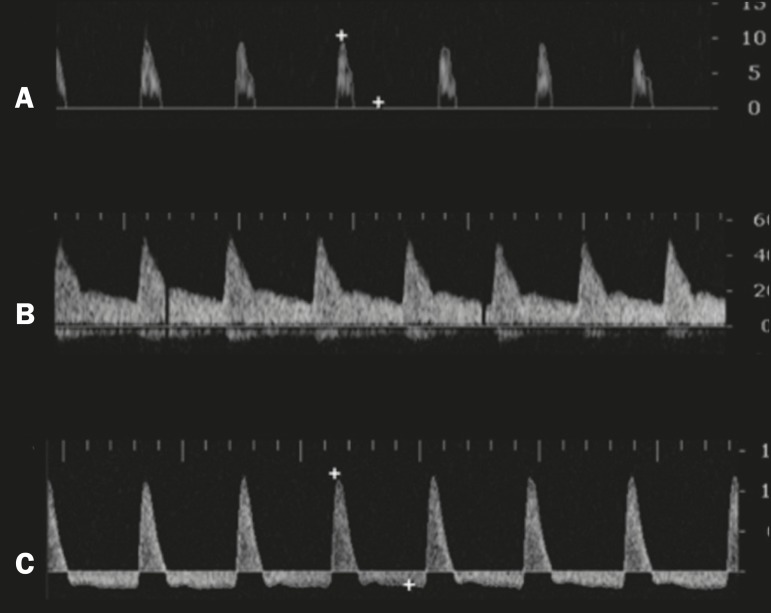
Graphs representing the color Doppler spectrum of the flow pattern of the cavernous arteries during the erection phases. **A:** Single-phase flow with minimal or absent diastole when the penis is flaccid. **B:** Increased systolic and diastolic flow 10 min after vasoactive drug injection. **C:** Increased systolic flow and reverse diastole 25 min after injection of prostaglandin.

**Figure 10:**
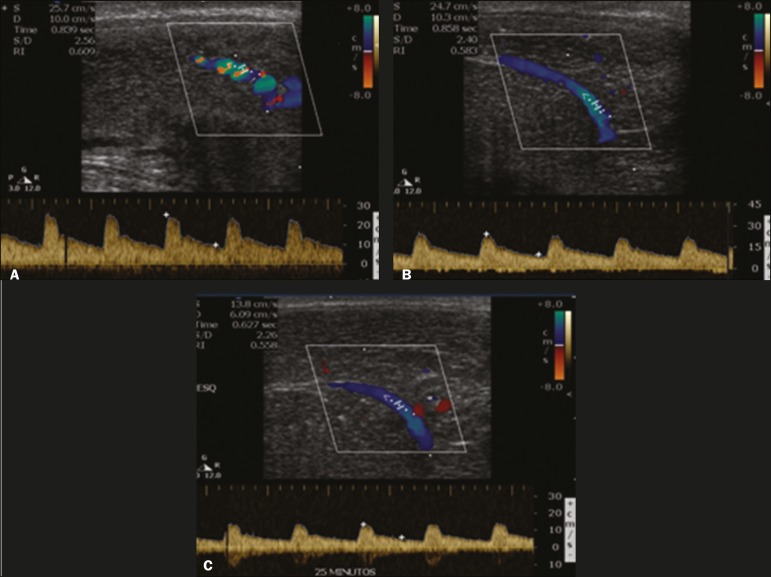
Longitudinal, ventral ultrasound of the penis, with pulsed mode and color Doppler. Flow of the cavernous arteries at 5, 15, and 25 min after prostaglandin injection (**A**, **B**, and **C**, respectively). Note that the cavernous artery flow remains below the expected levels (at least 25-35 cm/s), which indicates erectile dysfunction due to arterial insufficiency.

Prior to the injection of the chosen drug, the flow pattern is monophasic, with low systolic velocities and an absence of diastolic flow. After injection, it is expected that systolic and diastolic peak velocities will increase, decreasing progressively with vein occlusion and becoming negative when the penis becomes rigid (Figure 9). The reference values vary across studies, ranging from > 25 cm/s to > 35 cm/s^(11,12)^. Values above 35 cm/s indicate the absence of arterial disease, values below 25 cm/s indicate arterial insufficiency, and values of 25-35 cm/s are indeterminate because they are less specific (Figure 10). The data obtained should be correlated with the degree of erection observed. If the peak systolic velocities are normal, the final diastolic velocities should be evaluated, those above 5 cm/s being associated with venogenic erectile dysfunction.

## CONCLUSION

Ultrasound is a quite useful method, both for its availability and efficacy in penile evaluation: in terms of diagnosis, as in the case of penile fracture and Peyronie's disease; and in the identification of etiological factors, as in cases of priapism and erectile dysfunction. The knowledge of how to differentiate between normal and pathological aspects of the penis, as well as of how to manage Doppler techniques for this purpose, is extremely important for diagnosis and patient management.
